# The role of 24-h movement Behaviors in preschool children’s learning ability: the mediating effect of inhibitory control

**DOI:** 10.3389/fpsyg.2025.1673960

**Published:** 2025-11-19

**Authors:** Chang Zhenya, Huang Jia

**Affiliations:** 1 College of Preschool Education, Changsha Normal University, Changsha, China; 2 The First Beichen Kindergarten of Changsha Municipal Education Bureau, Changsha, China

**Keywords:** physical activity, sedentary behavior, sleep, inhibitory control, learning ability, movement behaviors

## Abstract

**Purpose:**

To investigate the impact of 24-h movement behaviors—sleep (SP), sedentary behavior (SB), light physical activity (LPA), and moderate-to-vigorous physical activity (MVPA)—on preschool children’s learning ability, with emphasis on inhibitory control as a mediating variable.

**Methods:**

Data were collected from 328 preschoolers (182 boys and 146 girls) using accelerometers and sleep questionnaires. Inhibitory control was assessed using the Early Years Toolbox, while learning ability was evaluated through the Learning Ability Test.

**Results:**

A 15-min daily increase in MVPA, replacing SP, SB, or LPA, was associated with improvements in preschoolers’ inhibitory control (0.038, 0.038, and 0.041, respectively) and learning ability (1.93, 1.87, and 2.52, respectively). Conversely, reallocating time in the opposite direction was associated with declines in both measures. Inhibitory control partially mediated the relationship between MVPA and learning ability across various demographic groups. For LPA, inhibitory control fully mediated its effect on learning ability in girls, while it fully mediated the impact of SP and MVPA on learning ability in boys.

**Conclusions:**

These findings collectively highlight the importance of 24-h movement behaviors in enhancing learning ability in preschool children through improved inhibitory control.

## 1 Introduction

The “Guidelines for Learning and Development of Children Aged 3–6” (2012), issued by the Ministry of Education of the People’s Republic of China, clearly state that children’s learning should be based on direct experience, primarily carried out through play and daily life activities. This indicates that appropriate learning methods for preschool children include “learning by doing,” “learning through play,” and “learning through experience,” among others. The ultimate goal of these activities is to promote the comprehensive development of children in cognitive, emotional, social, and physical domains (Piaget, 1952; Vygotsky, 1978). Through suitable learning approaches, children can not only establish a solid foundation for learning but also receive essential support in developing their learning abilities (Pellegrini, 2009). Preschool children’s learning abilities have a profound impact on their subsequent academic performance and achievements in later schooling (Campbell et al., 2001). Therefore, considering the multidimensional influence of early childhood learning abilities, adopting an interdisciplinary perspective for comprehensive research is particularly important. In this study, learning ability refers to cognitive skills in specific domains, such as language and mathematics (Shen, 2019; Yan, 2017; Zhu and Liu, 2019). The composite score of these abilities serves as a quantitative indicator of a child’s overall learning ability level.

In the field of sports science, researchers typically examine the effects of exercise (Fang, 2020; Hua et al., 2022; Zhang et al., 2021a), physical activity (PA) (Xu, 2022; Zhang et al., 2021b; Zhou et al., 2021), physical fitness (Cheng et al., 2021; Liang and Li, 2020; Liao et al., 2022), and 24-h movement behaviors (24 h-MBs) (Hinkley et al., 2020; Watson et al., 2020) on children’s learning ability or academic performance. These studies have consistently demonstrated a positive correlation between these factors and children’s academic performance.

In psychology, researchers typically examine the impact of cognitive functions, particularly executive function (EF) and self-regulation (SR), on children’s learning ability and academic performance. Numerous studies have highlighted that EF in early childhood can predict the development of learning ability throughout the school years and even into later life (Bachman et al., 2022; Waters et al., 2021). Self-regulation has also been shown to influence the development of early learning ability, including vocabulary and numerical concepts (McClelland et al., 2007; McClelland and Cameron, 2011).

In the field of early childhood education, Approaches to Learning have garnered increasing attention and have been incorporated into the learning standards and curriculum guidelines for preschool children in many countries (Office of Head Start, 2020; Department for Education, 2023; Australian Government Department of Education, 2022). In China, the “Guidelines for Learning and Development of Children Aged 3–6,” issued by the Ministry of Education in 2012, emphasize the importance of cultivating children’s Approaches to Learning. Consequently, scholars have focused on the positive association between Approaches to Learning and preschool children’s learning ability (Yang and Cai, 2022; Zhang, 2021).

From the perspective of developmental systems theory (Lerner, 2006; Sameroff, 2010), a child’s learning ability is not the product of any single factor but emerges from the dynamic interactions within a complex system encompassing biological, behavioral, and cognitive domains. This theoretical framework necessitates a holistic research design that moves beyond examining variables in isolation. Integrating the aforementioned fields, the variables influencing children’s learning ability encompass six main factors: exercise, PA, physical fitness, 24 h-MBs, EF, self-regulation, and approaches to learning. These variables can be categorized into the following three aspects.

PA and 24 h-MBs form the behavioral foundation of learning ability. PA, defined as bodily movements produced by skeletal muscle contractions that expend energy beyond basal metabolic levels (Ministry of Health, Bureau of Disease Prevention and Control, People’s Republic of China, 2012), encompasses various intensities, including exercise, which involves planned and structured activities. In recent years, the 24 h-MBs framework has emerged as a comprehensive approach that considers the interplay between sleep, sedentary behavior (SB), and PA across a continuum of intensities (Chang and Wang, 2020). This framework offers a more comprehensive understanding of how these behaviors collectively influence learning ability compared to a sole focus on PA. While physical fitness, comprising components such as body composition, cardiorespiratory fitness, muscular endurance, strength, and flexibility (Zhang and Fang, 2016), is a crucial research indicator, targeting it directly to enhance learning ability can be challenging. This is because significant fitness improvements often require intensive, specialized training and are subject to genetic constraints. In contrast, the 24 h-MBs framework is more readily translatable into practice. By focusing on the balance of sleep, SB, and PA throughout the day—behaviors that are naturally integrated into daily life—it provides a more systematic, holistic, and actionable foundation for optimizing children’s learning ability and developing feasible, evidence-based intervention strategies.

EF and SR serve as cognitive supports for learning ability. EF, a set of higher-order cognitive abilities encompassing working memory, inhibitory control (IC), and cognitive flexibility (Diamond, 2013), is often used as a mediating variable to explain the indirect effects of PA and physical fitness on academic performance (Liao et al., 2022; Wen et al., 2018; Zhang et al., 2021b). SR, a multi-faceted capacity involving the control of cognition, emotions, and behavior (McClelland and Cameron, 2011), is a concept with a complex relationship to EF. The conceptual relationship between SR and EF is viewed differently across theoretical perspectives. Some frameworks treat them as distinct constructs with clear boundaries: EF is typically defined as relatively pure ‘cool’ cognitive processes primarily involving prefrontal cortex-mediated cognitive control, whereas SR is conceptualized as broader ‘hot’ cognitive abilities encompassing the regulation of emotion, motivation, and behavior in social contexts (Zhou et al., 2012). However, an alternative integrative view posits that EF—particularly its core components such as IC—provides the foundational cognitive architecture upon which broader, emotionally and socially oriented SR is built (Barkley, 2012). Given that this study focuses specifically on IC as a core cognitive process, we adopt the latter integrative perspective, positioning IC as a fundamental mechanism underlying both EF and SR.

A synergistic relationship exists between Approaches to Learning and learning ability, connecting process with outcomes. Approaches to Learning encompass an individual’s attitudes, habits, emotional traits, and behavioral characteristics during the learning process, including enthusiasm, initiative, focus, persistence, creativity, and cooperation (Feng and Wang, 2023). While Approaches to Learning emphasize the process of learning (i.e., how one learns), learning ability focuses on the outcomes (i.e., what can be learned). Despite their distinct connotations, the two are intimately connected. Research indicates that Approaches to Learning often serve as crucial precursors to the development of learning ability in early childhood (Yang and Cai, 2022; Zhang, 2021). However, the assessment of learning dispositions lacks systematic standardized tools and relies primarily on contextualized subjective judgments, which leads to insufficient generalizability and comparability, thereby constraining the external validity and practical translation value of related research findings (Huo et al., 2024).

While the individual relationships between some of these variables have been explored, research that integrates them holistically remains scarce. For instance, it has been established that Approaches to Learning mediate the relationship between EF and early academic abilities (Zhang, 2021). Similarly, EF has been identified as a mediator in the effects of both physical fitness (Liao et al., 2022; Wen et al., 2018) and PA (Zhou et al., 2021) on academic performance. Other scholars have undertaken systematic reviews to map the broader connections among these constructs. Vabø et al. (2022), for example, explored the interrelationships among PA, self-regulation, EF, and learning ability, finding significant, albeit modest, associations. Ma et al. (2022), in their comprehensive review, confirmed the positive effects of PA interventions on EF and academic skills like literacy and mathematics, while also noting that the underlying mechanisms are not yet fully understood.

However, a significant gap persists. With the emergence of the 24 h-MBs framework—which provides a holistic view of sleep, SB, and PA across the day—research has begun to examine its link with EF in preschoolers (Bezerra et al., 2021; Lau et al., 2024; Lu et al., 2023). Yet, studies investigating the relationship between 24 h-MBs and learning ability or academic performance are still relatively limited (Hinkley et al., 2020). Crucially, no study to date has systematically investigated the intricate interplay among 24 h-MBs, IC, and learning ability within a single, cohesive model. This gap is particularly pressing given that the 24 h-MBs framework offers a more comprehensive and actionable foundation for developing real-world interventions than studying isolated behaviors like PA alone.

**Figure 1:**
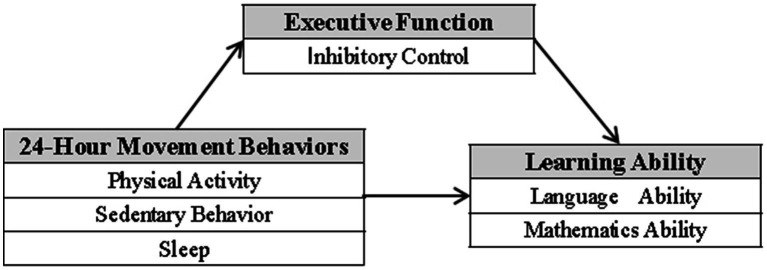
Hypothesized research model. This figure presents the theoretical model proposed in this study, depicting the hypothesized pathways among 24-h movement behaviors, inhibitory control, and learning ability. Model Components: Independent variables: Three components of 24-h movement behaviors (Sleep, Sedentary Behavior, Physical Activity). Mediating variable: Inhibitory Control. Dependent variable: Learning Ability (including both Language Ability and Mathematical Ability). Pathway Relationships: The model hypothesizes that 24-h movement behaviors both directly affect learning ability and indirectly affect it through the mediating role of inhibitory control.

Therefore, by integrating these distinct but related fields, the present study aims to address this void. It seeks to investigate the interrelationships among 24 h-MBs, IC, and learning ability in Chinese preschool children (Figure 1). Specifically, this study will address the following research questions:

1. Is there a significant association between preschool children’s 24 h-MBs and IC?
2. Is there a significant association between preschool children’s 24 h-MBs and learning ability?
3. Does IC mediate the relationship between 24 h-MBs and learning ability?

Furthermore, existing literature suggests potential gender differences in children’s activity patterns (Hinkley et al., 2008), cognitive development, and academic performance (Gestsdottir et al., 2014), though findings in preschool-aged children remain inconsistent. Given the lack of consistent prior evidence to form a strong *a priori* hypothesis, this study did not posit specific, directional hypotheses for gender differences. Instead, gender difference analysis was included as an exploratory aim to provide more context-specific evidence in this area and to inform future hypothesis-driven research.

## 2 Materials and methods

### 2.1 Subjects

This study adopted a cross-sectional design to recruit preschool children and their parents from one public kindergarten and two private kindergartens in Changsha City through convenience sampling. Participants were included if their parents provided informed consent, they exhibited no physical developmental disorders, and they did not have intellectual disabilities or cognitive disorders. The recruitment procedures, informed consent forms, testing protocols, and emergency safety plans for this study were approved by the Human Subjects Protection Committee (HR342-2024). The initial recruitment yielded 371 participants. During data collection, 43 participants were excluded from the final analysis due to either incomplete assessment completion or invalid data. Specifically, this included: Failure to complete one or more components of the 24 h-MBs assessment (e.g., insufficient accelerometer wear time); Incomplete IC or learning ability tests (e.g., child non-compliance or tester error); Missing or inconsistent responses in parent questionnaires. The final analyzable sample therefore consisted of 328 participants (182 males, 146 females) (Table 1). No data imputation was performed for missing values; only complete cases were included in the analysis.

**Table 1:** Demographic characteristics of the study participants (*N* = 328).

Variable	*N*	%
Sex		
Boy	182	55.49%
Girl	146	44.51%
Age (years)		
4	238	72.56%
5–6	90	27.44%

This table presents the basic demographic composition of the final analyzable sample. The sample consisted of 328 preschool children recruited from one public and two private kindergartens in Changsha City. As shown, there was a relatively even distribution by sex, with boys comprising slightly more than half of the sample (55.49%). The age distribution was notably skewed, with 4-year-olds constituting the majority (72.56%) of participants. This demographic profile is important for contextualizing the study’s findings and should be considered when interpreting the results, particularly regarding the generalizability to older preschool children.

### 2.2 Accelerometer

PA and SB were objectively measured using tri-axial accelerometers (ActiGraph GT3X-BT, Pensacola, FL, USA) that have been validated and widely used in PA research with preschool populations (Chang et al., 2020). Data collection was conducted over seven consecutive days (including five weekdays and two weekend days) to capture habitual activity patterns. Prior to deployment, all caregivers and preschool teachers received detailed “Accelerometer Usage Instructions” emphasizing the importance of continuous wear except during water-based activities and sleep.

Accelerometer data were processed using established protocols. The Choi algorithm (Choi et al., 2011) was applied to identify non-wear time, and data were collected at 15-s epochs to capture the sporadic activity patterns characteristic of young children. To be included in the analysis, participants were required to provide a minimum of three valid days of data (including at least two weekdays and one weekend day) with at least 480 min of valid wear time per day. PA intensities were classified using validated thresholds for Chinese preschool children (Chang and Wang, 2021): SB (0–116 counts/15 s), light physical activity (117–551 counts/15 s), moderate physical activity (552–997 counts/15 s), and vigorous physical activity (≥998 counts/15 s).

### 2.3 Sleep questionnaire

The sleep questionnaire comprised three key questions: (1) the typical bedtime in the past month; (2) the usual wake-up time in the past month; and (3) the number and duration of daytime naps, along with the total daytime sleep duration, over the past month (Chang and Wang, 2023).

The questionnaire differentiates between weekdays and weekends, providing predefined options for key time periods or durations (e.g., 6:00–10:00, 20:00–23:00, daytime 1–3 h), with 30-min intervals for the former and 60-min intervals for the latter. Actual sleep time was calculated based on the midpoint of each selected time interval. For example, if a parent reported their child’s sleep period as 20:00–21:00, the midpoint (20:30) would be used in subsequent calculations. Sleep duration is then computed using the following formula (Chang and Wang, 2020):


$$\text{Sleep Duration}=[\begin{array}{l} (\begin{array}{l} \text{Weekday Daytime Sleep Duration}+\\ \text{Weekday Nighttime Sleep Duration} \end{array})\times 5\\ +(\begin{array}{l} \text{Weekend Daytime Sleep Duration}+\\ \text{Weekend Nighttime Sleep Duration} \end{array})\times 2 \end{array}]/7$$

To mitigate potential recall bias, we optimized the questionnaire format by employing predefined time intervals, provided detailed guidance to parents on referencing daily records or digital tools, and applied a weighted formula to calculate weekly average sleep duration. These strategies collectively enhance the accuracy of sleep data while minimizing recall bias (Chang and Wang, 2020, 2023).

### 2.4 Learning ability test

The learning ability assessment, a cognitive or academic ability test, was adapted from Tsinghua University’s “China Urbanization and Children Development Survey” (CUCDS). Designed by Professor Zhang Houcan from Beijing Normal University for Chinese children aged 3–12, this test is suitable for the age range of participants in this study (Shen, 2019; Yan, 2017; Zhu and Liu, 2019). Specifically, language and mathematics ability tests designed for 3-6-year-olds were employed. The language test assessed vocabulary, memory, comprehension, and knowledge, while the mathematics test focused on calculation and reasoning skills. While learning ability encompasses a broader range of cognitive skills, language, and mathematics are commonly used proxies in previous research and exhibit strong reliability, with coefficients consistently exceeding 0.8 (Shen, 2019; Yan, 2017).

### 2.5 Inhibitory control test

The Early Years Toolbox (EYT), developed by Steven Howard’s team at the University of Wollongong, was employed to assess early childhood EF. This tool, applicable to preschool children aged 4 and older and early primary school students (Howard and Melhuish, 2017; Qu et al., 2020), is well-suited for this age group. The EYT Fish-Shark (Go/No-Go) task, specifically targeting IC, was selected for this study, aligning with previous research (Bezerra et al., 2021; Lu et al., 2023). This focus on IC is justified by several factors: first, the interconnected nature of EF components (IC, working memory, and cognitive flexibility) in preschool children makes their isolation challenging (Diamond, 2016); second, a factor analysis study by Wiebe et al. (2008) demonstrated the effectiveness of IC as a representative of EF in this age group; and third, the significant time commitment (approximately 45 min) and high dropout rate (nearly 50%) associated with assessing all three components simultaneously in preschool children (Bezerra et al., 2021) make it impractical for large-scale studies.

### 2.6 Socio-economic status questionnaire

The Socio-economic Status (SES) questionnaire, adapted from Yuan et al. (2009), assesses parental education levels, occupations, and family income. SES is calculated using the methodology established by the Programme for International Student Assessment (PISA) (OECD, 2003) and categorized into three levels based on standard deviations. Occupational classification is based on the International Socio-Economic Index (ISEI) scoring system (Ganzeboom and Treiman, 1996).

### 2.7 Procedure

Phase One: Thirty preschool children and their parents were randomly selected from the sample and assessed using a triaxial accelerometer, a learning ability test, a sleep questionnaire, and an SES survey. A pilot test was conducted to refine the implementation plan for the formal study and ensure its scientific validity and ethical compliance. Informed consent was obtained from all participating children’s parents prior to the study.

Phase Two: Formal testing commences with a 24 h-MBs assessment utilizing a three-axis accelerometer to objectively measure preschool children’s PA and SB. Concurrently, sleep and SES data were collected via online questionnaires. Subsequent to the 24 h-MBs assessment, IC and learning ability tests were administered. The testing procedure involved the following steps: (1) One day prior to testing, the research team distributed answer record sheets to class teachers for the completion of children’s basic information; (2) On the testing day, record sheets were distributed to participating children, who brought them to the designated testing site. Children were tested in groups of five under the supervision of research staff or class teachers; (3) Each child underwent IC and learning ability tests, with each test session lasting approximately 25 min; (4) Upon completion, record sheets were collected and stored by research staff; (5) Children were returned to their classrooms by research staff or teachers. To ensure data quality, tests were administered in a quiet, well-lit environment with individual supervision. Children provided verbal responses to questions. The testing duration was strictly controlled: 20 min, with 10 min allocated to each of the language and mathematics sections. Time limits were strictly enforced. In contrast, IC tests allowed for flexible timing, taking 6.8 min on average (with a range of 5 to 8 min), enabling children to complete tasks at their own pace.

### 2.8 Data analysis

First, to examine potential gender differences in all study variables (24 h-MBs, IC, learning ability, and their subdomains), independent samples t-tests were employed for normally distributed data, while the Mann–Whitney U test was used for variables violating normality assumptions. Results for parametric tests are presented as mean ± standard deviation (x̄ ± SD). Second, bivariate Pearson correlations were conducted to assess the preliminary associations among the core variables. Second, to address Research Questions (1) and (2), three complementary analytical methods were employed to investigate the relationships between 24 h-MBs, IC, and learning ability from different perspectives. Bivariate Pearson correlations were conducted to preliminarily explore pairwise associations among the variables; Compositional data analysis (using R 4.3.1) was performed, employing isometric log-ratio transformations in regression models to examine the combined effects of the 24 h-MBs components on IC and learning ability; Isotemporal substitution analysis (Dumuid et al., 2019; Dumuid et al., 2018), was used to investigate how reallocating time between different movement behaviors might predict changes in IC and learning ability. Finally, to address Research Question (3), path analysis was conducted using AMOS 24.0 to test the mediating role of IC in the relationship between 24 h-MBs and learning ability (Wu, 2010), In this model, learning ability was specified as a latent variable, while all other variables, including IC and 24 h-MBs, were treated as observed variables. The analysis controlled for gender, age, and SES.

## 3 Results

### 3.1 Basic characteristics of the study population

Boys exhibited significantly lower levels of SB than girls (*t* = −3.210, *p* = 0.001). Conversely, boys demonstrated significantly higher levels of light physical activity (LPA) (*t* = 4.075, *p* < 0.001) and moderate-to-vigorous physical activity (MVPA) (*t* = 7.753, *p* < 0.001) than girls. Additionally, boys scored significantly lower on IC assessments compared to girls (*t* = −3.192, *p* = 0.002).

Children aged 4 years exhibited significantly higher levels of sleep (*t* = 4.636, *p* < 0.001) and SB (*t* = 2.490, *p* = 0.013) compared to those aged 5–6 years. Conversely, they displayed significantly lower levels of MVPA (*t* = −2.647, *p* = 0.009). In terms of cognitive abilities, the 4-year-old group scored significantly lower than the 5–6 year age group in IC (*t* = −5.385, *p* < 0.001), language ability (*t* = −6.584, *p* < 0.001), and mathematical ability (*t* = −6.110, *p* < 0.001).

Regarding socioeconomic status, children from low-SES and middle-SES backgrounds exhibited significantly higher sleep duration (*F* = 4.511, *p* = 0.012) and MVPA levels (*F* = 5.972, *p* = 0.003) compared to those from high-SES backgrounds. Furthermore, children from low-SES backgrounds engaged in significantly more MVPA than those from middle-SES backgrounds. In contrast, high-SES children demonstrated significantly superior language ability (*F* = 5.077, *p* = 0.007) and mathematical ability (*F* = 9.077, *p* < 0.001) compared to both middle-SES and low-SES groups. Additionally, middle-SES children exhibited significantly higher mathematical ability compared to low-SES children (Table 2).

**Table 2:** Differences in 24-h movement behaviors, inhibitory control, and cognitive abilities across demographic groups.

Variable	Group	*N*	24-h movement behaviors (min/d)				Inhibitory control	Learning ability (standard score)	
			SP	SB	LPA	MVPA		Language ability	Mathematical ability
Sex	Boy	182	658.27 ± 34.81	471.61 ± 95.26^**^	221.46 ± 37.54^***^	58.73 ± 18.08^***^	0.70 ± 0.20^**^	100.61 ± 14.35	102.56 ± 15.80
	Girl	146	658.11 ± 30.43	507.15 ± 104.92^**^	206.02 ± 31.07^***^	44.49 ± 15.35^***^	0.77 ± 0.18^**^	100.43 ± 13.63	99.33 ± 14.04
Age	4	238	663.22 ± 31.29^***^	494.45 ± 109.82^*^	212.86 ± 35.02	50.76 ± 17.70^**^	0.70 ± 0.20^***^	97.58 ± 12.57^***^	97.85 ± 13.09^***^
	5–6	90	644.92 ± 33.47^***^	468.87 ± 70.27^*^	219.16 ± 36.89	56.70 ± 19.30^**^	0.82 ± 0.18^***^	108.32 ± 14.69^***^	109.77 ± 16.65^***^
SES	Low(a)	26	669.89 ± 46.92^a > c^	476.46 ± 68.72	217.62 ± 32.04	61.77 ± 18.58^a > b^	0.71 ± 0.19	95.49 ± 12.67^a < c^	91.77 ± 12.72^a < b^
	Middle(b)	232	659.57 ± 29.78^b > c^	487.78 ± 111.43	213.83 ± 35.74	52.77 ± 18.42^b > c^	0.72 ± 0.21	99.85 ± 13.87^b < c^	100.71 ± 14.68^b < c^
	High(c)	70	649.29 ± 34.94	490.36 ± 71.31	215.99 ± 36.76	47.66 ± 16.50	0.77 ± 0.16	104.64 ± 14.16	105.95 ± 15.60
Overall		328	658.20 ± 32.88	487.43 ± 101.08	214.59 ± 35.60	52.39 ± 18.32	0.73 ± 0.20	100.53 ± 14.01	101.12 ± 15.10

This table presents descriptive statistics (Mean ± Standard Deviation) for 24-h movement behaviors, inhibitory control, and cognitive abilities, stratified by sex, age, and socioeconomic status (SES). SP = Sleep; SB = Sedentary Behavior; LPA = Light Physical Activity; MVPA = Moderate-to-Vigorous Physical Activity; SES = Socioeconomic Status. In the SES classification, a represents low SES, b represents middle SES, and c represents high SES. *p < 0.05, **p < 0.01, and ***p < 0.001.

It should be noted that the age distribution of our sample was uneven, with a high proportion of 4-year-olds. This characteristic may have influenced the results. For instance, compared to a sample with a more balanced age distribution, the overall average activity levels reported here might be more representative of younger children, while the advantages in cognitive and academic abilities observed in older children (aged 5–6 years) might be amplified. This sample characteristic should be considered when interpreting the findings.

### 3.2 Correlations among 24 h-MBs, IC, and learning ability in preschool children

To address Research Questions (1) and (2), which explore the fundamental relationships between 24 h-MBs, IC, and learning ability, we first conducted correlation analyses. Significant correlations were observed among various parameters.

SB was negatively correlated with MVPA (*r* = −0.290, *p* < 0.001). LPA exhibited significant positive correlations with MVPA (*r* = 0.618, *p* < 0.001), IC (*r* = 0.135, *p* = 0.014), and mathematical ability (*r* = 0.124, *p* = 0.024). MVPA showed significant positive correlations with both IC (*r* = 0.187, *p* = 0.001) and mathematical ability (*r* = 0.206, *p* < 0.001). IC was positively correlated with language ability (*r* = 0.162, *p* = 0.003) and mathematical ability (*r* = 0.252, *p* < 0.001) (Table 3).

**Table 3:** Correlations among 24-h movement behaviors, inhibitory control, and learning ability in preschool children.

Variable	SP	SB	LPA	MVPA	Inhibitory control	Language ability
SP	---					
SB	0.063	---				
LPA	−0.099	−0.090	---			
MVPA	−0.088	−0.290^***^	0.618^***^	---		
Inhibitory control	0.011	−0.034	0.135^*^	0.187^**^	---	
Language ability	0.003	0.002	0.078	0.107	0.162^**^	---
Mathematical ability	−0.024	−0.072	0.124^*^	0.206^***^	0.252^***^	0.418^***^

This table displays Pearson correlation coefficients among the core study variables, including 24-h movement behavior components, inhibitory control, and learning ability measures. SP = Sleep; SB = Sedentary Behavior; LPA = Light Physical Activity; MVPA = Moderate-to-Vigorous Physical Activity. Interpretation Guide: Correlation Coefficients: Values range from −1 to +1, indicating the strength and direction of linear relationships. Positive values indicate positive relationships, negative values indicate inverse relationships. Significance Marks: *p < 0.05, **p < 0.01, and ***p < 0.001.

### 3.3 Assessment of the impact of 24 h-MBs on IC and learning ability after controlling for confounders

To further address Research Questions (1) and (2), we employed compositional data regression and isotemporal substitution analysis, controlling for confounding factors, to examine the specific effects and substitution relationships of 24 h-MBs on IC and learning ability.

After controlling for confounding factors such as gender, age, and SES, MVPA remained significantly associated with IC (γ_1_ = 0.17 [0.07, 0.27], *p* = 0.001), mathematical ability (γ_1_ = 10.47 [3.45, 17.49], *p* = 0.004), and overall learning ability (γ_1_ = 9.28 [2.69, 15.88], *p* = 0.006) (Table 4).

**Table 4:** Associations between 24-h movement behaviors, inhibitory control, and learning ability using compositional data analysis.

Dependent variable	SP		SB		LPA		MVPA	
	γ_1_^1^	*p*	γ_1_^2^	*p*	γ_1_^3^	*p*	γ_1_^4^	*p*
Inhibitory control	−0.20	0.145	−0.13	0.296	−0.08	0.439	0.17	0.001
Language ability	0.37	0.969	−2.68	0.760	−5.90	0.435	4.20	0.228
Mathematical ability	1.25	0.898	−2.23	0.805	−12.59	0.105	10.47	0.004
Learning ability	1.04	0.909	−2.82	0.739	−11.56	0.113	9.28	0.006

This table presents results from compositional data regression analyses, showing the independent associations of each movement behavior with outcome variables while controlling for the overall 24-h movement behaviors composition (i.e., accounting for the interdependence among components). The γ coefficients represent the expected change in the outcome variable when the relative proportion of a specific behavior in the 24-h composition increases. SP = Sleep; SB = Sedentary Behavior; LPA = Light Physical Activity; MVPA = Moderate-to-Vigorous Physical Activity. Interpretation Guide: The γ coefficient indicates the strength of association between a relative increase in the specific movement behavior within the 24-h composition and the outcome variable score, while keeping the relative proportions of other behaviors constant. For example, the relative proportion of MVPA is positively associated with inhibitory control scores (γ = 0.17, *p* = 0.001).

Drawing on previous research (Bezerra et al., 2021), an analysis was conducted using an isochronous substitution of 15 min per day as an example. The findings revealed that when MVPA replaced sleep, SB, or LPA by 15 min per day, preschool children’s IC scores increased by 0.038, 0.038, and 0.041, respectively. Conversely, replacing MVPA with sleep, SB, or LPA led to decreases of 0.051, 0.051, and 0.053, respectively. Similarly, when MVPA replaced sleep, SB, or LPA, learning ability scores increased by 1.93, 1.87, and 2.52, respectively. Conversely, replacing MVPA with sleep, SB, or LPA resulted in decreases of 2.61, 2.55, and 3.61, respectively. Likewise, mathematical ability scores increased by 2.16, 2.10, and 2.79, respectively, when MVPA replaced sleep, SB, or LPA. Conversely, replacing MVPA with these behaviors led to decreases of 2.92, 2.86, and 3.51, respectively (Table 5).

**Table 5:** Changes in inhibitory control and learning ability following 15-minute isotemporal substitution in 24-h movement behaviors.

+15 min	SP	SP	SP	SB	SB	SB	LPA	LPA	LPA	MVPA	MVPA	MVPA
-15 min	SB	LPA	MVPA	LPA	MVPA	SP	SB	MVPA	SP	SB	LPA	SP
Inhibitory control	−0.001	0.002	−0.051*	0.003	−0.051*	0.001	−0.002	−0.053*	−0.002	0.038*	0.041*	0.038*
Language ability	−0.06	0.29	−1.24	0.35	−1.18	0.06	−0.33	−1.51	−0.27	0.86	1.22	0.92
Mathematical ability	−0.06	0.64	−2.92*	0.70	−2.86*	0.06	−0.65	−3.51*	−0.59	2.10*	2.79*	2.16*
Learning ability	−0.07	0.58	−2.61*	0.65	−2.55*	0.07	−0.61	−3.16*	−0.54	1.87*	2.52*	1.93*

This table presents results from isotemporal substitution models, showing the predicted changes in inhibitory control and academic abilities when 15 min are reallocated from one movement behavior to another within the 24-h composition. SP = Sleep; SB = Sedentary Behavior; LPA = Light Physical Activity; MVPA = Moderate-to-Vigorous Physical Activity. Interpretation Guide: Value Interpretation: The values represent the expected change in outcome variables when increasing the behavior in the “+15 min” column by 15 min while simultaneously decreasing the behavior in the “–15 min” column by 15 min. Positive values indicate expected improvements, while negative values indicate expected declines. * Indicates *p* < 0.05.

### 3.4 Mediating effect of IC in the relationship between 24 h-MBs and learning ability

This study directly tested Research Question (3), The study employed structural equation modeling (SEM) to assess the model fit across the overall sample of preschool children and various sub-samples (boys, girls, low-to-middle SES children, and high SES children). Model fit was evaluated using various fit indices, as detailed in Table 5. Results indicate that the CMIN values were relatively small and non-significant for both the overall sample and the different sub-samples. Additionally, CMIN/DF values were below 2, suggesting a good model fit. The CFI and TLI values exceeded 0.85, falling within the acceptable range. The RMSEA values were less than 0.05 for the overall sample, girls, low-to-middle SES children, and high SES children, indicating excellent model fit. For boys, the RMSEA value was below 0.08, which is also considered acceptable (Table 6).

**Table 6:** Model fit indices for the overall sample and subsamples.

Group	*N*	CMIN	DF	CMIN/DF	P	TLI	CFI	RMSEA
Overall	328	6.422	5	1.284	0.267	0.979	0.995	0.029
Boy	182	10.168	5	1.218	0.071	0.866	0.968	0.076
Girl	146	6.488	5	1.422	0.262	0.955	0.989	0.045
Low-to-Middle SES	258	6.283	5	1.257	0.280	0.974	0.994	0.032
High SES	70	5.726	5	1.145	0.334	0.965	0.992	0.046

This table presents the goodness-of-fit indices for the structural equation model testing the mediating role of inhibitory control in the relationship between 24-h movement behaviors and learning ability, evaluated across the overall sample and key demographic subsamples. SES = Socio-economic status. Interpretation Guide: CMIN/DF (χ^2^/df): Values between 1 and 3 indicate acceptable model fit. CFI and TLI: Values > 0.90 suggest acceptable fit, while values > 0.95 indicate excellent fit. RMSEA: Values < 0.08 represent acceptable fit, and values < 0.05 indicate excellent fit.

**Figure 2:**
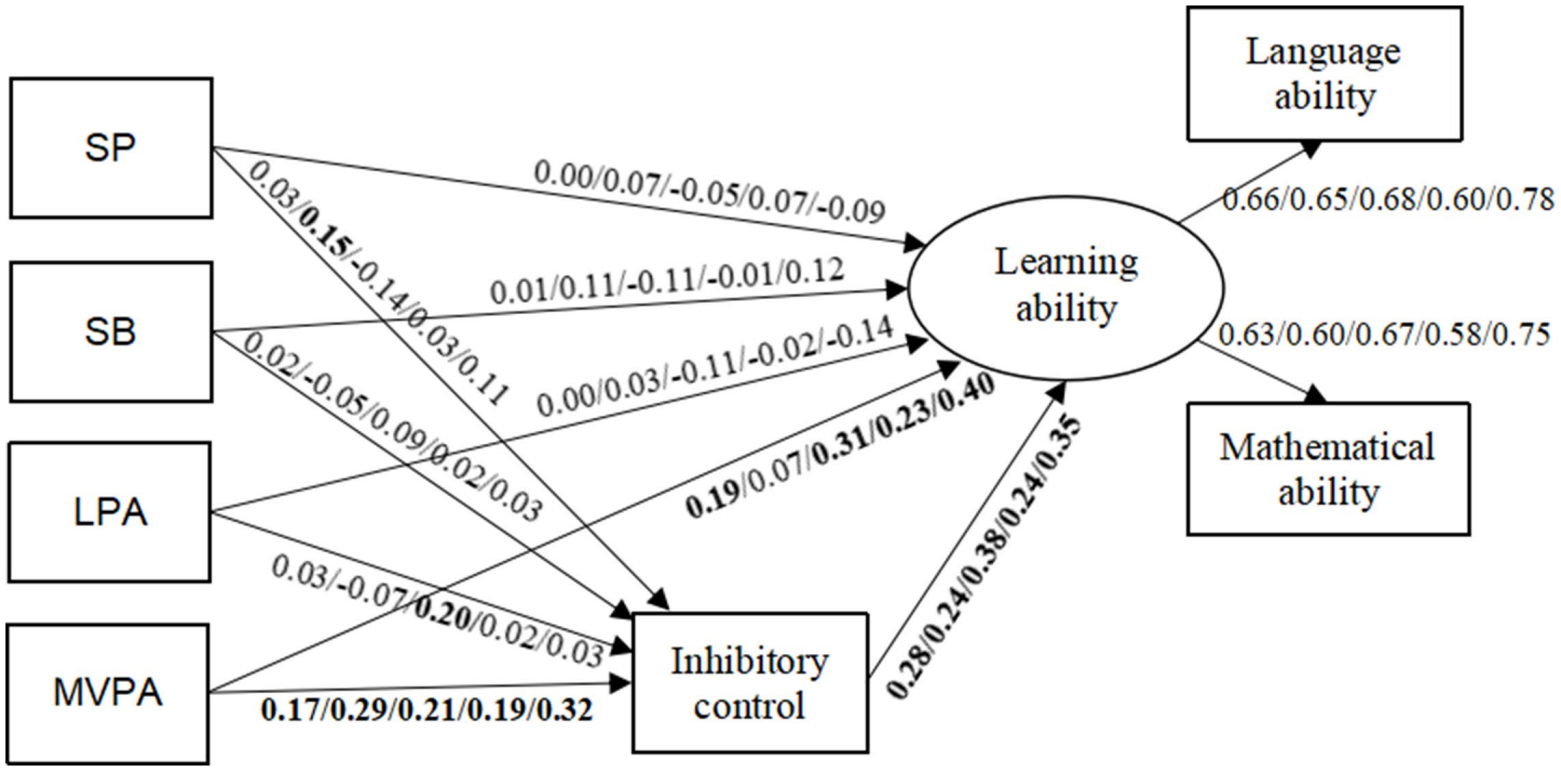
Path diagram of the impact of 24-h movement behaviors on preschool children’s learning ability and the underlying mechanisms. This figure displays the results of the path analysis based on empirical data, presenting standardized path coefficients for the overall sample and different subgroups (boys, girls, low-to-middle SES children, high SES children). SP = Sleep; SB = Sedentary Behavior; LPA = Light Physical Activity; MVPA = Moderate-to-Vigorous Physical Activity; SES = Socioeconomic Status. Interpretation Guide: The values shown in the figure represent standardized path coefficients, indicating the strength and direction of relationships between variables. Statistically significant paths (*p* < 0.05) across all subgroup analyses are highlighted in bold.

The mediation analysis results indicated that for girls, low-to-middle SES children, high SES children, and the overall sample, MVPA positively influenced IC (*E* = 0.17, 0.21, 0.19, and 0.32, respectively, *p* < 0.05) and learning ability (*E* = 0.19, 0.31, 0.23, and 0.40, respectively, *p* < 0.05). Furthermore, IC positively impacted learning ability (*E* = 0.28, 0.38, 0.24, and 0.35, respectively, *p* < 0.05). Therefore, IC serves as a partial mediator in the relationship between MVPA and learning ability across these groups (Figure 2).

In the sample of girls, LPA positively affected IC (*E* = 0.20, *p* < 0.05), which in turn positively influenced learning ability (*E* = 0.38, *p* < 0.05). However, LPA did not exhibit a significant direct association with learning ability (*E* = −0.11, *p* > 0.05). Therefore, IC fully mediates the relationship between LPA and learning ability in girls.

In the sample of boys, both sleep (*E* = 0.15, *p* < 0.05) and MVPA (*E* = 0.29, *p* < 0.05) positively impacted IC, which, in turn, positively affected learning ability (*E* = 0.24, *p* < 0.05). However, neither sleep (*E* = 0.07, *p* > 0.05) nor MVPA (*E* = 0.07, *p* > 0.05) was directly correlated with learning ability. This suggests that IC fully mediates the relationship between sleep, MVPA, and learning ability in boys.

Across all SES subgroups, MVPA consistently demonstrated a positive ICfect on IC (*E* = 0.21/0.19/0.32, *p* < 0.05) and learning ability (*E* = 0.31/0.23/0.40, *p* < 0.05). Furthermore, IC significantly influenced learning ability in all subgroups (*E* = 0.38/0.24/0.35, *p* < 0.05). These results indicate that IC consistently served as a partial mediator in the relationship between MVPA and learning ability across different socioeconomic strata.

## 4 Discussion

This study adopts a multidisciplinary perspective grounded in developmental systems theory to investigate the relationship between preschool children’s 24 h-MBs and learning ability, emphasizing the mediating role of IC. Our findings, which reveal the interconnected effects of behavioral (24 h-MBs) and cognitive (IC) systems on learning outcomes, underscore the theory’s premise that development arises from multi-level, dynamic interactions (Lerner, 2006; Sameroff, 2010). The results provide a scientific foundation for optimizing children’s movement behaviors and for designing effective health intervention strategies.

### 4.1 The association between preschool children’s 24 h-MBs and IC

In recent years, research on the relationship between 24 h-MBs and IC has gradually increased, generally revealing a positive association between MVPA and IC. The findings of this study further confirm this relationship (Bezerra et al., 2021; Lau et al., 2024; Yin et al., 2024; Xu et al., 2024). However, some studies have reached different conclusions (Lu et al., 2023), with one study finding no significant association between MVPA and IC (measured by the EYT—Fish-Shark Test). Two factors may account for this discrepancy. First, discrepancies in tools and methodologies may significantly influence the results. A key contextual factor is that children’s PA often varies between weekdays and weekends (Zhang et al., 2020). Lu et al. (2023) used a 5-day wear protocol, which likely missed a full weekend cycle and thus may not fully capture these habitual activity patterns. In contrast, this study and most related research employed a 7-day protocol, which includes a complete weekend and provides a more comprehensive and representative picture of children’s activity behaviors. Second, differences in sample size and confounding variables could also influence the results. Lu et al. (2023) had a relatively small sample of 135 participants, whereas this study had a larger sample and controlled for potential confounders, such as SES, thereby enhancing the generalizability and applicability of the findings.

Regarding the relationship between LPA and IC in preschool children, the results show considerable divergence across studies. This inconsistency may be partly explained by the nature of the IC measures used. As highlighted in the literature, performance-based psychometric tests (such as the Fish-Flanker or Fish-Shark tasks) and caregiver-reported questionnaires (such as the BRIEF-P) are known to capture distinct aspects of IC, with the latter often providing a more ecologically valid assessment of complex, everyday behaviors (Barkley, 2012; Soto et al., 2020; Toplak et al., 2013). For instance, some studies found no significant association between LPA and IC when measured by the Fish-Flanker task (Yin et al., 2024), whereas others reported a significant negative correlation when using the BRIEF-P questionnaire (Xu et al., 2024). Additionally, a significant positive correlation was observed between LPA and IC as measured by the Early Years Toolbox (Fish-Shark Test) (Bezerra et al., 2021). In the present study, LPA showed a significant positive correlation with IC in general data analysis, but this association was no longer significant when LPA was analyzed as component data. This pattern not only underscores the sensitivity of analytical methods in handling LPA data but also highlights how the choice of IC measure—questionnaire versus performance test—may lead to differing conclusions regarding the relationship between LPA and IC in young children.

In summary, the inconsistencies in research findings on the relationship between 24 h-MBs and IC primarily stem from differences in analytical methods, sample representativeness, and measurement tools. This study further confirms the significant association between MVPA and IC, contributing to the existing body of knowledge and addressing Research Question (1). Future studies should strive for greater consistency in research design and methodological approaches to enhance the comparability and reliability of results.

### 4.2 The association between preschool children’s 24 h-MBs and learning ability

This study revealed a significant association between LPA and MVPA within 24 h-MBs and mathematical ability, but not language ability, addressing Research Question 2. These findings aligned with previous research. Scholars investigating the relationship between PA and early academic learning have identified a significant correlation between PA and early mathematical skills, rather than vocabulary expression (Vabø et al., 2022). Literature suggests that the association between PA and mathematical ability can be attributed to several factors: (1) Mathematics often involves spatial reasoning and geometric concepts, which PA can enhance by improving spatial perception and orientation (Morawietz and Muehlbauer, 2021). For example, movement can help children understand object size, shape, and spatial relationships. Research has indicated that children with more developed fundamental motor skills perform better in mathematics due to superior performance on specific spatial ability tasks (Scott et al., 2024). (2) Mathematics requires precise logical thinking and problem-solving skills, which are also exercised through sports activities, such as strategizing and making quick decisions in team sports (Singh et al., 2012). (3) The interaction between motor skills and mathematical skills may contribute to this association. Some studies suggest a positive correlation due to shared processing mechanisms in the brain (Kang et al., 2023). For example, research has found that Chinese students exhibit notable brain activity in motor regions during Arabic mathematical addition and size comparison tasks (Tang et al., 2006).

### 4.3 The mediating role of IC in the relationship between 24 h-MBs and learning ability

Our study supported previous findings that PA, especially MVPA, was significantly associated with learning ability (Vabø et al., 2022; Zhou et al., 2021). It further revealed the mediating role of IC in this relationship. Specifically, IC partially mediated the relationship between MVPA and learning ability in the overall sample, girls, children from low-to-middle SES households, and children from high-SES households. In contrast, IC fully mediated this relationship in boys.

Besides, the present study found that IC fully mediated the relationship between LPA and learning ability in girls, but no mediating effect was observed in boys. This discrepancy might be attributed to the fact that boys generally engage in significantly higher levels of PA than girls (Chang et al., 2023; Wang et al., 2023), making the impact of LPA less significant for boys. However, for girls, who typically exhibited lower activity levels, even LPA plays a crucial role in improving their IC and learning ability. Related research has shown that over the past two decades, PA has had a positive impact on EF and academic performance in school-aged children (6–12 years old), with the most significant effects observed through several weeks of regular PA interventions (Yan et al., 2020). Furthermore, scholars have suggested that MVPA may indirectly enhance academic performance through EF, highlighting that MVPA interventions focused on developing EF may be particularly beneficial for academic achievement (Zhou et al., 2021). The findings of this study aligned with these observations.

Finally, this study revealed that IC fully mediated the relationship between sleep duration and learning ability in boys. While numerous studies have demonstrated a significant association between sleep and cognitive development (Xing et al., 2018; Zhou et al., 2013), this study indicated that this association was particularly pronounced in male preschoolers. Conversely, no significant relationship between sleep and learning ability was observed in girls, which might be attributed to the generally lower sleep quality among female preschoolers (Xing et al., 2018). Future research should further explore the underlying mechanisms of this gender difference.

In summary, this study systematically addresses Research Question 3 by uncovering the significant impact of 24 h-MBs on learning ability in preschool children and the mediating role of IC. By simultaneously examining interrelated factors within subgroups (e.g., male vs. female, varying SES levels), the study explored potential heterogeneity in these associations. This approach not only captures overall effects but also highlights subgroup-specific patterns, providing valuable insights for targeted interventions.

### 4.4 Research limitations

This study has several limitations that should be addressed in the future. Firstly, EF is typically divided into three components: inhibition, shifting, and updating. However, this study only assessed inhibition. While previous research has provided justification for this choice (Bezerra et al., 2021), this limitation may affect the comprehensiveness of EF assessments and potentially skew the examination of its mediating role. Secondly, the cross-sectional design of this study limits the ability to analyze causal relationships among 24 h-MBs, IC, and learning ability. Longitudinal studies predicting learning ability based on 24 h-MBs during preschool years would provide more informative insights. Thirdly, this study measured PA intensity using accelerometers. However, these devices have limitations in capturing specific activity types (e.g., rolling, crawling, climbing, or cycling) or distinguishing between different types of SB (Vabø et al., 2022). Studies indicate inconsistent cognitive associations with different types of SB (Li et al., 2022). Therefore, future studies should consider using methods such as direct observation or activity diaries to more accurately classify activity types and further explore their contributions to learning abilities.

## 5 Conclusion

Twenty-four hour MBs in preschool children significantly impact their learning ability, with MVPA playing a particularly prominent role. This impact is partially or fully mediated by IC. These findings provide a scientific foundation for optimizing children’s movement behavior patterns and developing effective health promotion strategies. Thus, for educators and caregivers, it is recommended to prioritize MVPA in daily schedules—for instance, by replacing SB or LPA with structured aerobic games and motor skill exercises. For parents, establishing consistent sleep routines and reducing screen-based SB can further support cognitive development. Through these approaches, we can effectively leverage the synergistic benefits of 24-MBs to provide robust support for learning readiness and cognitive growth in preschool-aged children.

## Data Availability

The raw data supporting the conclusions of this article will be made available by the authors, without undue reservation.
